# Anti-oxidative stress regulator NF-E2-related factor 2 mediates the adaptive induction of antioxidant and detoxifying enzymes by lipid peroxidation metabolite 4-hydroxynonenal

**DOI:** 10.1186/2045-3701-2-40

**Published:** 2012-11-28

**Authors:** Ying Huang, Wenge Li, Ah-Ng Tony Kong

**Affiliations:** 1 Department of Pharmaceutics, Center for Cancer Prevention Research, Ernest Mario School of Pharmacy, Rutgers, the State University of New Jersey, 160 Frelinghuysen Road, Piscataway, NJ, 08854, USA; 2 Present address: Department of Genetics, Albert Einstein College of Medicine, 1301 Morris Park Avenue, Bronx, NY, 10461, USA

**Keywords:** NRF2, 4-hydroxynonenal (4-HNE), Antioxidant response element (ARE), Oxidative stress

## Abstract

**Background:**

NF-E2-related factor 2 (NRF2) regulates a battery of antioxidative and phase II drug metabolizing/detoxifying genes through binding to the antioxidant response elements (*ARE*). NRF2-*ARE* signaling plays a central role in protecting cells from a wide spectrum of reactive toxic species including reactive oxygen/nitrogen species (RONS). 4-hydroxylnonenal (4-HNE) is a major end product from lipid peroxidation of omega-6 polyunsaturated fatty acids (PUFA) induced by oxidative stress, and it is highly reactive to nucleophilic sites in DNA and proteins, causing cytotoxicity and genotoxicity. In this study, we examined the role of NRF2 in regulating the 4-HNE induced gene expression of antioxidant and detoxifying enzymes.

**Results:**

When HeLa cells were treated with 4-HNE, NRF2 rapidly transloated into the nucleus, as determined by the distribution of NRF2 tagged with the enhanced green fluorescent protein (EGFP) and increased NRF2 protein in the nuclear fraction. Transcriptional activity of *ARE*-luciferase was significantly induced by 0.01-10 μM of 4-HNE in a dose-dependent manner, and the induction could be blocked by pretreatment with glutathione (GSH). 4-HNE induced transcriptional expression of glutathione S-transferase (GST) A4, aldoketone reductase (AKR) 1C1 and heme oxygenase-1 (HO-1), and the induction was attenuated by knocking down *NRF2* using small interfering RNA.

**Conclusions:**

NRF2 is critical in mediating 4-HNE induced expression of antioxidant and detoxifying genes. This may account for one of the major cellular defense mechanisms against reactive metabolites of lipids peroxidation induced by oxidative stress and protect cells from cytotoxicity.

## Background

Polyunsaturated fatty acids (PUFA), essential components of cell membrane, are susceptible to oxidation initiated by free radicals [1]. 4-hydroxylnonenal (4-HNE) is an end product from lipid peroxidation of omega-6 (*n*-6) PUFA [2]. The physiological concentration of 4-HNE is generally at the low micromolar level, but is remarkably increased under continuous oxidative stress [3]. As an α,β-unsaturated aldehyde, 4-HNE is highly reactive to a variety of nucleophilic sites in DNA and proteins [4]. Exposure to excessive 4-HNE can cause cytotoxicity, inactivation of enzymes, redox imbalance and activation of multiple signaling events, and 4-HNE is implied in the detrimental pathogenesis of a number of degenerative diseases including cancer [5,6]. Several metabolic pathways are involved in the detoxification of 4-HNE, including conjugation with glutathione (GSH) catalyzed by glutathione S-transferases (GST) and reduction of the aldehyde group to corresponding alcohol by aldoketone reductases (AKR) [5].

Eukaryotic cells have developed highly efficient machineries to counteract oxidative stress from environmental insults and aerobic metabolisms [7]. Antioxidant response elements (*ARE*) are identified in the regulatory region of many cytoprotective genes that encode phase II drug metabolizing/detoxifying enzymes, antioxidant enzymes and phase III transporters [8,9]. When oxidative stress is elevated, NF-E2-related factor 2 (NRF2) will be activated to trigger gene expression through binding to *ARE*[10]. Subsequently, it leads to enhanced cellular capability to remove excess electrophiles and restore redox homeostasis [11].

NRF2 activity is regulated in part by a repressor protein, Kelch-like ECH-associated protein 1 (KEAP1), which retains NRF2 in the cytoplasm and mediates its degradation under homeostatic conditions [12]. Stimuli such as dietary antioxidants, heavy metals and reactive oxygen species (ROS) can disrupt the NRF2-KEAP1 binding and induce nuclear translocation of NRF2 where it dimerizes with small Maf proteins and binds to *ARE*[12]. In addition, some studies have shown that the subcellular distribution of NRF2 can also be controlled by the net driving force of nuclear location signals (NLS) and nuclear export signals (NES) [13] and phosphorylation of NRF2 [7] .

In this study, we investigated the role of NRF2 in regulating the gene expression of antioxidant and detoxifying enzymes upon the exposure to 4-HNE. Our results show that NRF2 rapidly translocates into nucleus after exposure to 4-HNE and induces transcriptional activity of *ARE* and mRNA expressions of *AKR1C1*, *GSTA4* and heme oxygenase-1 (*HO-1*). The induction of these detoxifying enzymes is diminished when *NRF2* is knocked down using small interfering RNA.

## Results

### NRF2 translocates into the nucleus after 4-HNE treatment

**Figure 1:**
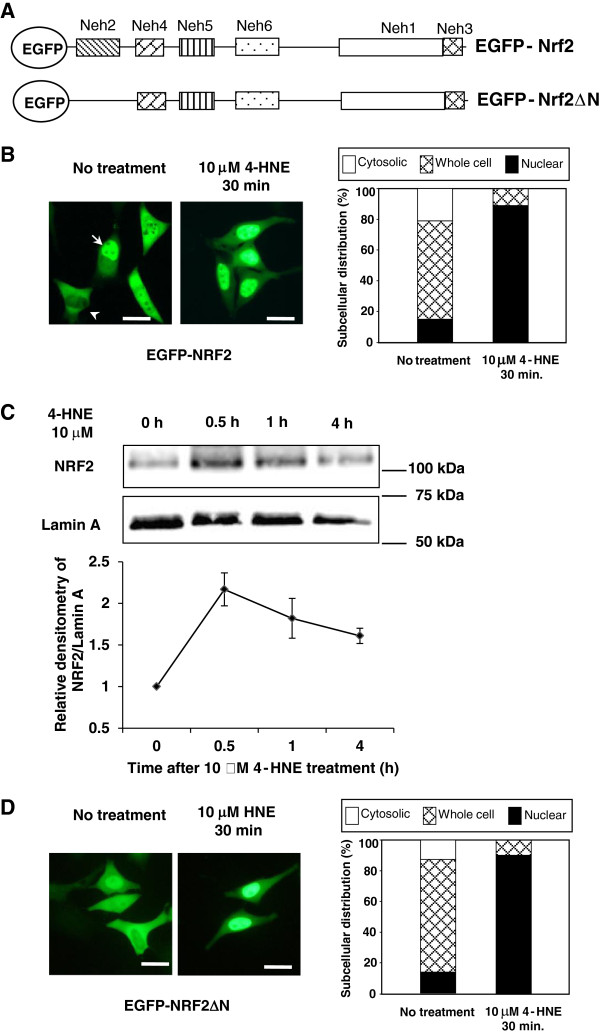
**4-HNE treatment induces NRF2 nuclear translocation.** (**A**) The schematic diagram showing the structures of NRF2 constructs used in the study. (**B**) Under the untreated condition, EGFP-NRF2 exhibited a mixed pattern of nuclear (arrow), whole cell and cytosolic (arrowhead) distribution. After treatment of 10 μM 4-HNE for 30 min, a predominantly nuclear distribution was observed. Scale bar: 10 μm. (**C**) Time course of nuclear NRF2 accumulation after 10 μM 4-HNE treatment. *: *P* < 0.05. (**D**) Deletion of the KEAP1-binding domain (NRF2ΔN) did not affect 4-HNE induced nuclear translocation of NRF2.

When expressed in HeLa cells, NRF2 tagged with the enhanced green fluorescent protein (EGFP) exhibited a heterologous distribution pattern (Figure 1B). Cell percentage assay showed that 64% of cells exhibited a whole cell distribution pattern. About 15% of cells showed a nuclear distribution (Figure 1B, arrow) and 21% of cells showed a cytosolic distribution (Figure 1B, arrowhead). After treatment with 10 μM 4-HNE for 30 min, nearly 90% of cells exhibited a nuclear distribution (Figure 1B), indicating robust nuclear translocation of NRF2.

To further confirm the nuclear translocation effect elicited by 4-HNE, we examined the nuclear NRF2 protein level by Western blotting analyses. HeLa cells were treated with 10 μM 4-HNE for 0, 0.5,1 and 4 h. 4-HNE markedly elevated the nuclear NRF2 protein level, with the highest accumulation at 0.5 h after treatment (Figure 1C). Similar effects have been observed in PC12 cells and vascular endothelial cells [14,15].

Next, the N-terminal truncation mutant of NRF2 (EGFP-NRF2ΔN, Figure 1D) lacking the KEAP1 domain, was expressed in HeLa cells. Under the unstressed condition, EGFP-NRF2ΔN demonstrated a heterologous distribution pattern (Figure 1D). Treatment with 10 μM 4-HNE (30 min) converted the distribution of EGFP-NRF2ΔN to a predominant nuclear pattern (Figure 1D). Previous study showed that no NRF2ΔN/KEAP1 binding was detected when NRF2ΔN was co-expressed with KEAP1 [16]. Since NRF2ΔN is free from KEAP1 sequestration in the cytosol, the NRF2ΔN distribution can be deemed as free floating in the cell. These results show that 4-HNE can directly affect the subcellular distribution of NRF2 into the nucleus.

### *ARE*-luciferase activity increases after 4-HNE treatment

**Figure 2:**
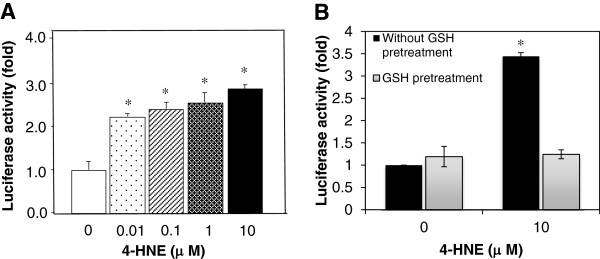
***ARE*-luciferase activity significantly increases by 4-HNE treatment.** (**A**) HeLa cells were transiently transfected with *ARE*-luciferase construct for 24 h and treated with 0.01-10 μM 4-HNE. Cells were harvested and lysed after 6 h, and luciferase activity was measured. (**B**) HepG2 cells stably expressed with *ARE*-luciferase reporter gene were treated with 10 μM 4-HNE with or without 2 h pretreatment of 5 mM GSH. Cells were harvested and lysed after 18 h, and luciferase activity was measured. Relative fold of induction was obtained as compared to the untreated cells. *: *P* < 0.05.

To determine whether the nuclear accumulation of NRF2 could increase the transcriptional activity of *ARE*, we co-expressed 0.5 μg pcDNA3.1-*NRF2* with 0.25 μg *ARE*-luciferase reporter in HeLa cells. Twenty-four hours after transfection, cells were treated with 0, 0.01, 0.1, 1, 10 μM 4-HNE for 30 min and then cultured in fresh medium for 6 h. HNE treatments elicited significant *ARE*-luciferase inductions in a dose-dependent manner (Figure 2A).

HepG2-C8 cells with stably expressed p*ARE*-TI-luciferase constructs [17] were treated with 0 and 10 μM 4-HNE for 18 h. *ARE*-luciferase activity was significantly induced with 10 μM 4-HNE treatment compared with the untreated cells (Figure 2B). Since the conjugation to GSH is the major metabolism pathway for 4-HNE, it will result in a net loss of intracellular GSH and redox imbalance. We found that when cells were pretreated with 5 mM GSH, the induction of *ARE*-luciferase activity was completely blocked (Figure 2B).

### NRF2 is critical in regulating the expression of detoxifying genes

**Figure 3:**
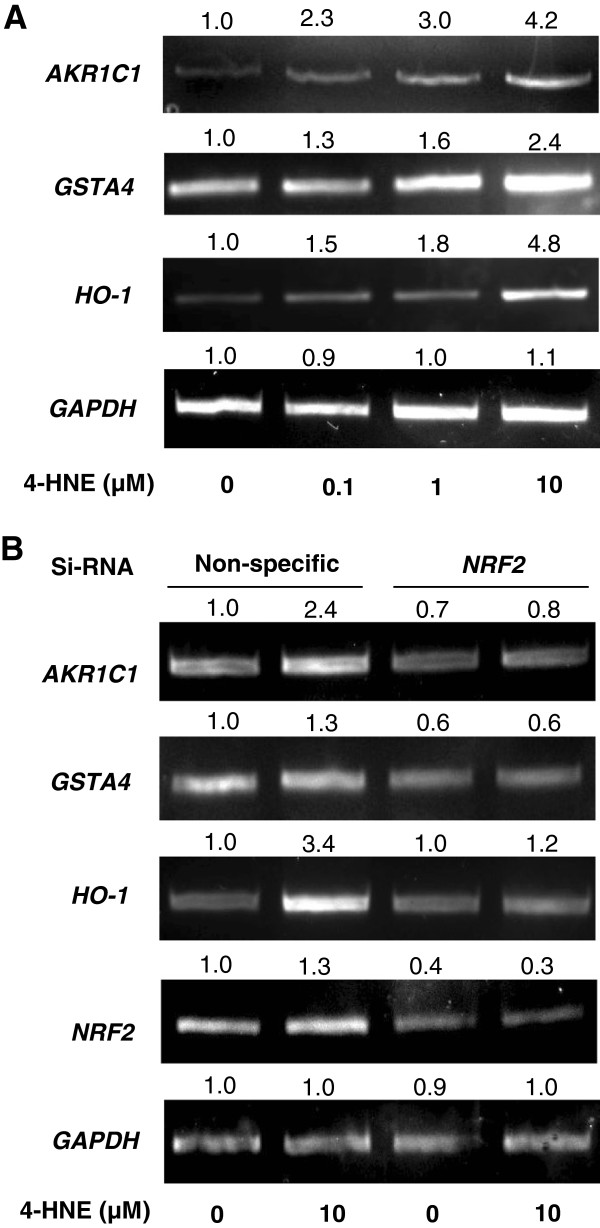
**Effects of NRF2 on the mRNA expression of metabolizing and antioxidant enzymes.** (**A**) HeLa cells were treated with 0, 0.1, 1 and 10 μM of 4-HNE for 6 h. mRNA expression of *AKR1C1*, *GSTA4* and *HO-1* was induced by 4-HNE in a dose-dependent manner. (**B**) HeLa cells were transfected with *NRF2* specific or non-specific siRNA for 24 h, followed by 4-HNE treatment for 6 h. Knocking-down of NRF2 attenuated the induction of *AKR1C1*, *GSTA4* and *HO-*1by 4-HNE. Densitometry analyses of the PCR products were performed by Image J program, and the numbers at the top of each band are relative density compared to the control.

When HeLa cells were treated with 4-HNE, in agreement with the enhanced *ARE*-luciferase activity, RT-PCR results showed that 4-HNE induced the transcription of *AKR1C1, GSTA4* and *HO-1* in a dose-dependent manner (Figure 3A). To examine whether the induction of these detoxifying and antioxidant genes was dependent on NRF2, HeLa cells were transfected with *NRF2* specific siRNA or nonspecific siRNA as a control. *NRF2* expression was down-regulated by the siRNA transfection (Figure 3B). In the cells transfected with control siRNA, the expression of *AKR1C1, GSTA4* and *HO-1* was markedly induced by 10 μM 4-HNE, while in the cells transfected with *NRF2* siRNA, the induction of these genes was attenuated (Figure 3B).

## Discussion

It is well recognized that our body has a comprehensive antioxidant system to counter oxidative stress. 4-HNE, an oxidative stressor, causes adaptive induction of detoxifying enzymes such as AKR1C1 and GSTA4 in different cell lines [18,19], although the molecular pathway is not fully understood. The common feature of these detoxifying genes is that they have *ARE*-like sequences in their 5’-flanking regions [20,21]. In this study, we demonstrate that NRF2 mediates 4-HNE induced gene expression of key antioxidant and detoxifying enzymes, resulting in enhanced 4-HNE metabolism.

4-HNE is a highly reactive electrophile, and several studies have reported that it is a potent NRF2 inducer [14,15]. Our current results further confirmed that the nuclear translocation of NRF2 is significantly increased by 10 μM 4-HNE treatment in HeLa cells (Figure 1). There are several mechanisms proposed for the 4-HNE-induced NRF2 activation. 4-HNE can react with cysteine sites in the KEAP1 protein and that may disrupt the KEAP1-dependent degradation of NRF2 [22]. In addition, 4-HNE may activate NRF2 through activation of upstream kinases such as protein kinase C, extracellular signal-regulated protein kinase and phosphoinositide 3-kinase [14,23,24]. In this study, we showed that 4-HNE could induce nuclear translocation of the NRF2 mutant lacking the KEAP1 binding domain (Figure 1D), indicating that 4-HNE may have a direct effect on NRF2 itself. We found that 4-HNE could modify NRF2 protein in an *in vitro* testing system (Additional file 1: Figure S1), and future studies will be needed to identify the specific amino acid sites modified by 4-HNE and their impacts in NRF2 signaling. In addition, several studies have reported that 4-HNE treatment leads to dramatic decrease of intracellular GSH [25,26], and depletion of GSH can activate NRF2 signaling [27]. In the present study, we showed that pretreatment of GSH could block the induction of ARE transcriptional activity by 4-HNE (Figure 2B), suggesting that 4-HNE may activate NRF2 via depletion of GSH.

GSTA4 and AKR1C1 are two important enzymes for detoxification of 4-HNE. In the large GST family, GSTA4 is the most active isoform in catalyzing conjugation of GSH to 4-HNE [4]. The higher expression level of GSTA4 in DU145 prostate cancer cells is associated with faster 4-HNE metabolism rate, compared to PC3 or LNCaP prostate cancer cells [28]. It is also reported that overexpression of GSTA4 protects HepG2 cells from 4-HNE mediated oxidative injuries [29]. AKR1C1 has high catalytic activity in reducing 4-HNE to less toxic 1,4-dihydroxynonenol [19]. The role of HO-1 in the detoxification of 4-HNE is not clear. The induction of HO-1 may enhance the overall cellular antioxidant capacity and prevent oxidative stress induced cytotoxicity [15]. Therefore, the induction of gene expression of these cellular protective enzymes by 4-HNE appears to be an adaptive response to enhance elimination of 4-HNE and reduce its toxicity. The transcriptional induction of these detoxifying and antioxidant genes is attenuated when *NRF2* is knocked down (Figure 3C), indicating that the induction is mediated by NRF2.

## Conclusions

In this study, we demonstrate that NRF2 regulates the enhanced gene expression of antioxidant and detoxifying enzymes by 4-HNE. Our study highlights the importance of the NRF2-*ARE* signaling mechanism in the detoxification of reactive lipid metabolites such as 4-HNE.

## Methods

### Cell culture and chemicals

Human cervical squamous cancerous HeLa cells and human hepatoma HepG2 cells were obtained from ATCC (Manassas, VA). The establishment of stably expressed HepG2 cells with the ARE luciferase reporter was described previously [17]. Cells were cultured in Dolbecco’s modified eagle medium supplemented with 10% FBS. 4-HNE was purchased from Cayman Chemical (Ann Arbor, Michigan).

### Cell fractionation and Western blotting

HeLa cells were treated with 10 μM of 4-HNE for 0, 0.5, 1 and 4 h, and then rinsed with ice-cold PBS and harvested. Nuclear protein was extracted using NE-PER nuclear and cytoplasmic protein extraction kits (Thermo scientific) according to the manufacturer’s instruction. The protein concentration of each sample was measured, and 10 μg of nuclear proteins were used for Western blotting analyses. The details of Western blotting procedures were described previously [30]. Antibodies against NRF2 and Lamin A were from Epitomics and Santa Cruz, respectively. The densitometry of the bands were analyzed by ImageJ program.

### Epifluorescent Microscopy

HeLa cells were cultured on ethanol-sterilized glass coverslips and transfected with 1 μg of EGFP-NRF2 or its EGFP-NRF2ΔN using the Lipofectamine method (Invitrogen) and further cultured in DMEM for 24 h. The generation of plasmids was described in our previous study [30]. After transfection, cells were treated with 10 μM of 4-HNE for 30 min. The expression and subcellular distribution of EGFP-tagged NRF2 and NRF2ΔN were examined using a Nikon Eclipse E600 epifluorescent microscope and a Nikon C-SHG1 UV light source purchased from Micron-Optics (Cedar Knolls, NJ). The EGFP signals were examined using FITC filters. The epifluorescent images were digitalized using the Nikon DXM1200 camera and Nikon ACT-1 software.

### Luciferase Activity Assay

The HepG2-C8 cell line with a stably expressed p*ARE*-TI-luciferase construct was previously established [17] and used to test the ARE transcriptional activity in this study. Cells were seeded in 6-well plates overnight, and then treated with 10 μM of 4-HNE for 18 h with or without pretreatment of 5 mM glutathione (GSH) for 2 h. Cells were then washed twice with ice-cold PBS and lysed with 1× reporter lysis buffer (Promega). A 10 μl lysate was mixed with the luciferase substrate (Promega) and the luciferase activity was measured using a Sirius luminometer (Berthold Detection System) and normalized by protein concentration.

### Reverse Transcription-PCR

RNA was extracted using RNeasy mini kit (Qiagen, Valencia, CA) according to manufacturer’s instructions and reverse transcribed (RT) using TaqMan® reverse transcription reagents (Applied Biosystems). The RT products were further analyzed by PCR reactions. The sequences of the PCR primers used are listed in Table 1. The RT-PCR products were resolved in 1.5% agarose gel with ethidium bromide and visualized in UV light.

**Table 1:** Oligonucleotide primers used for PCR

**Gene**	**NCBI ID**	**Primer sequence**
*GAPDH*	NM_002046.3	Forward 5’-AAGGTCGGAGTCAACGGATTTGGT-3’
		Reverse 5’- ACAAAGTGGTCGTTGAGGGCAATG-3’
*AKR1C1*	NM_001353	Forward 5’- AGCTTTGGTGCAATTCCCATCGAC-3’
		Reverse 5’- GGGCTTTGCTGTAGCTTGCTGAAA-3’
*GSTA4*	NM_001512.3	Forward 5’- TGAAGTTGGTACAGACCCGAAGCA-3’
		Reverse 5’- ACCATGACAGAGCTGGGATCCATT-3’
*HO-1*	NM_002133	Forward 5’- AGGAGATTGAGCGCAACAAGGAGA-3’
		Reverse 5’- TCGCCACCAGAAAGCTGAGTGTAA-3’

### Transfection with siRNA

The sense and antisense sequences of siRNA against *NRF2* and nonspecific sequences were described previously [31]. The siRNA oligomers were synthesized by Integrated DNA technologies. HeLa cells were transfected using Lipofectamin RNAiMAX reagent (Invitrogen) following the manufacturer’s instructions. Cells were transfected for 48 h with 50 nM siRNA in Opi-MEM medium without antibiotics and serum. Then, the cells were treated with 10 μM 4-HNE for 6 h.

### Statistical analyses

Fold induction of *ARE-*luciferase and relative densitometry of nuclear NRF2 protein were analyzed using one-way ANOVA, where 4-HNE concentration or the exposure time of 4-HNE was treated as the main effect, followed by Tukey’s studentized range test.

## Abbreviations

ARE: Antioxidant response elements; AKR1C1: Aldoketone reductase 1C1; EGFP: The enhanced green fluorescent protein; GSH: Glutathione; GSTA4: Glutathione S-transferase A4; HO-1: Heme oxygenase-1; 4-HNE: 4-hydroxynonenal; KEAP1: Kelch-like ECH-associated protein 1; NRF2: NF-E2-related factor 2.

## Competing interests

The authors declare that they have no competing interests.

## Authors’ contributions

YH and WL designed, conducted the experiments, analyzed the data and wrote the manuscript; ANK designed, supervised the experiments and edited the manuscript; ANK has the primary responsibility for the final content; all the authors read and approved the final version of the manuscript.

## Supplementary Material

Additional file 1 **Figure S1.** NRF2 protein is modified by 4-HNE. Purified 6×His-NRF2 protein (10 μg, 0.146 nmol) was incubated with different molar excess of 4-HNE in 30 μL phosphate buffer (50mM, pH = 7.4) for 30 min. Reaction was terminated by adding 10 μL of Laemmli's SDS buffer and samples were boiled at 95°C. 20 μL of each sample was subject to Western blotting analyses. Primary anti-4-HNE antibody (Alpha Diagnostic) was used to detect 4-HNE modifications. Then, the primary antibody was stripped off and anti-NRF2 antibody was used to detect NRF2 protein as a loading control. Click here for file

## References

[B1] Catala A Lipid peroxidation of membrane phospholipids generates hydroxy-alkenals and oxidized phospholipids active in physiological and/or pathological conditions Chem Phys Lipids 2009 157 1 11 10.1016/j.chemphyslip.2008.09.004 18977338

[B2] Esterbauer H Schaur RJ Zollner H Chemistry and biochemistry of 4-hydroxynonenal, malonaldehyde and related aldehydes Free Radic Biol Med 1991 11 81 128 10.1016/0891-5849(91)90192-6 1937131

[B3] Siems W Grune T Intracellular metabolism of 4-hydroxynonenal Mol Aspects Med 2003 24 167 175 10.1016/S0098-2997(03)00011-6 12892994

[B4] Awasthi YC Ansari GA Awasthi S Regulation of 4-hydroxynonenal mediated signaling by glutathione S-transferases Methods Enzymol 2005 401 379 407 16399399 10.1016/S0076-6879(05)01024-4

[B5] Petersen DR Doorn JA Reactions of 4-hydroxynonenal with proteins and cellular targets Free Radic Biol Med 2004 37 937 945 10.1016/j.freeradbiomed.2004.06.012 15336309

[B6] Smathers RL Galligan JJ Stewart BJ Petersen DR Overview of lipid peroxidation products and hepatic protein modification in alcoholic liver disease Chem Biol Interact 2011 192 107 112 10.1016/j.cbi.2011.02.021 21354120 PMC3109208

[B7] Jaiswal AK Nrf2 signaling in coordinated activation of antioxidant gene expression Free Radic Biol Med 2004 36 1199 1207 10.1016/j.freeradbiomed.2004.02.074 15110384

[B8] Xie T Belinsky M Xu Y Jaiswal AK ARE- and TRE-mediated regulation of gene expression. Response to xenobiotics and antioxidants J Biol Chem 1995 270 6894 6900 10.1074/jbc.270.12.6894 7896838

[B9] Shen G Kong AN Nrf2 plays an important role in coordinated regulation of Phase II drug metabolism enzymes and Phase III drug transporters Biopharm Drug Dispos 2009 30 345 355 10.1002/bdd.680 19725016 PMC2782863

[B10] Itoh K Chiba T Takahashi S Ishii T Igarashi K Katoh Y Oyake T Hayashi N Satoh K Hatayama I Yamamoto M Nabeshima Y An Nrf2/small Maf heterodimer mediates the induction of phase II detoxifying enzyme genes through antioxidant response elements Biochem Biophys Res Commun 1997 236 313 322 10.1006/bbrc.1997.6943 9240432

[B11] Kensler TW Wakabayashi N Nrf2: friend or foe for chemoprevention? Carcinogenesis 2010 31 90 99 10.1093/carcin/bgp231 19793802 PMC2802668

[B12] Motohashi H Yamamoto M Nrf2-Keap1 defines a physiologically important stress response mechanism Trends Mol Med 2004 10 549 557 10.1016/j.molmed.2004.09.003 15519281

[B13] Li W Kong AN Molecular mechanisms of Nrf2-mediated antioxidant response Mol Carcinog 2009 48 91 104 10.1002/mc.20465 18618599 PMC2631094

[B14] Chen ZH Saito Y Yoshida Y Sekine A Noguchi N Niki E 4-Hydroxynonenal induces adaptive response and enhances PC12 cell tolerance primarily through induction of thioredoxin reductase 1 via activation of Nrf2 J Biol Chem 2005 280 41921 41927 10.1074/jbc.M508556200 16219762

[B15] Ishikado A Nishio Y Morino K Ugi S Kondo H Makino T Kashiwagi A Maegawa H Low concentration of 4-hydroxy hexenal increases heme oxygenase-1 expression through activation of Nrf2 and antioxidative activity in vascular endothelial cells Biochem Biophys Res Commun 2010 402 99 104 10.1016/j.bbrc.2010.09.124 20920477

[B16] Katoh Y Iida K Kang MI Kobayashi A Mizukami M Tong KI McMahon M Hayes JD Itoh K Yamamoto M Evolutionary conserved N-terminal domain of Nrf2 is essential for the Keap1-mediated degradation of the protein by proteasome Arch Biochem Biophys 2005 433 342 350 10.1016/j.abb.2004.10.012 15581590

[B17] Yu R Mandlekar S Lei W Fahl WE Tan TH Kong AN p38 mitogen-activated protein kinase negatively regulates the induction of phase II drug-metabolizing enzymes that detoxify carcinogens J Biol Chem 2000 275 2322 2327 10.1074/jbc.275.4.2322 10644681

[B18] Raza H John A 4-hydroxynonenal induces mitochondrial oxidative stress, apoptosis and expression of glutathione S-transferase A4-4 and cytochrome P450 2E1 in PC12 cells Toxicol Appl Pharmacol 2006 216 309 318 10.1016/j.taap.2006.06.001 16843508

[B19] Burczynski ME Sridhar GR Palackal NT Penning TM The reactive oxygen species–and Michael acceptor-inducible human aldo-keto reductase AKR1C1 reduces the alpha, beta-unsaturated aldehyde 4-hydroxy-2-nonenal to 1,4-dihydroxy-2-nonene J Biol Chem 2001 276 2890 2897 10.1074/jbc.M006655200 11060293

[B20] Hayes JD Flanagan JU Jowsey IR Glutathione transferases Annu Rev Pharmacol Toxicol 2005 45 51 88 10.1146/annurev.pharmtox.45.120403.095857 15822171

[B21] Burczynski ME Lin HK Penning TM Isoform-specific induction of a human aldo-keto reductase by polycyclic aromatic hydrocarbons (PAHs), electrophiles, and oxidative stress: implications for the alternative pathway of PAH activation catalyzed by human dihydrodiol dehydrogenase Cancer Res 1999 59 607 614 9973208

[B22] Levonen AL Landar A Ramachandran A Ceaser EK Dickinson DA Zanoni G Morrow JD Darley-Usmar VM Cellular mechanisms of redox cell signalling: role of cysteine modification in controlling antioxidant defences in response to electrophilic lipid oxidation products Biochem J 2004 378 373 382 10.1042/BJ20031049 14616092 PMC1223973

[B23] Numazawa S Ishikawa M Yoshida A Tanaka S Yoshida T Atypical protein kinase C mediates activation of NF-E2-related factor 2 in response to oxidative stress Am J Physiol Cell Physiol 2003 285 C334 C342 12700136 10.1152/ajpcell.00043.2003

[B24] Chen J Wang L Chen Y Sternberg P Cai J Phosphatidylinositol 3 kinase pathway and 4-hydroxy-2-nonenal-induced oxidative injury in the RPE Invest Ophthalmol Vis Sci 2009 50 936 942 18806289 10.1167/iovs.08-2439 PMC2716057

[B25] Long EK Murphy TC Leiphon LJ Watt J Morrow JD Milne GL Howard JR Picklo MJ Sr Trans-4-hydroxy-2-hexenal is a neurotoxic product of docosahexaenoic (22:6; n-3) acid oxidation J Neurochem 2008 105 714 724 10.1111/j.1471-4159.2007.05175.x 18194211

[B26] Vladykovskaya E Sithu SD Haberzettl P Wickramasinghe NS Merchant ML Hill BG McCracken J Agarwal A Dougherty S Gordon SA Schuschke DA Barski OA O'Toole T D'Souza SE Bhatnagar A Srivastava S Lipid peroxidation product 4-hydroxy-trans-2-nonenal causes endothelial activation by inducing endoplasmic reticulum stress J Biol Chem 2012 287 11398 11409 10.1074/jbc.M111.320416 22228760 PMC3322871

[B27] Copple IM Goldring CE Jenkins RE Chia AJ Randle LE Hayes JD Kitteringham NR Park BK The hepatotoxic metabolite of acetaminophen directly activates the Keap1-Nrf2 cell defense system Hepatology 2008 48 1292 1301 10.1002/hep.22472 18785192

[B28] Pettazzoni P Ciamporcero E Medana C Pizzimenti S Dal Bello F Minero VG Toaldo C Minelli R Uchida K Dianzani MU Pili R Barrera G Nuclear factor erythroid 2-related factor-2 activity controls 4-hydroxynonenal metabolism and activity in prostate cancer cells Free Radic Biol Med 2011 51 1610 1618 10.1016/j.freeradbiomed.2011.07.009 21816220

[B29] Gallagher EP Huisden CM Gardner JL Transfection of HepG2 cells with hGSTA4 provides protection against 4-hydroxynonenal-mediated oxidative injury Toxicol In Vitro 2007 21 1365 1372 10.1016/j.tiv.2007.04.004 17553661 PMC2785086

[B30] Kim JH Yu S Chen JD Kong AN The nuclear cofactor RAC3/AIB1/SRC-3 enhances Nrf2 signaling by interacting with transactivation domains Oncogene 2012 Epub ahead of print 10.1038/onc.2012.59 PMC3538952 22370642

[B31] Singh A Misra V Thimmulappa RK Lee H Ames S Hoque MO Herman JG Baylin SB Sidransky D Gabrielson E Brock MV Biswal S Dysfunctional KEAP1-NRF2 interaction in non-small-cell lung cancer PLoS Med 2006 3 e420 10.1371/journal.pmed.0030420 17020408 PMC1584412

