# Supplementary figures and images for: Anti-oxidative stress regulator NF-E2-related factor 2 mediates the adaptive induction of antioxidant and detoxifying enzymes by lipid peroxidation metabolite 4-hydroxynonenal
Source: Cell Biosci. 2012 Nov 28;2:40. doi: 10.1186/2045-3701-2-40 (PMC3519783; doi:10.1186/2045-3701-2-40)

## Supplemental figure

Molar excess  
of 4-HNE

0X

5X

10X

25X

50X

4-HNE

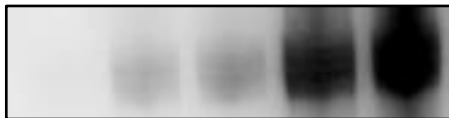

NRF2

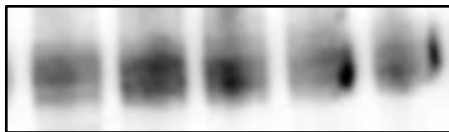

Supplement: Additional file 1 — Figure S1. NRF2 protein is modified by 4-HNE. Purified 6×His-NRF2 protein (10 μg, 0.146 nmol) was incubated with different molar excess of 4-HNE in 30 μL phosphate buffer (50mM, pH = 7.4) for 30 min. Reaction was terminated by adding 10 μL of Laemmli's SDS buffer and samples were boiled at 95°C. 20 μL of each sample was subject to Western blotting analyses. Primary anti-4-HNE antibody (Alpha Diagnostic) was used to detect 4-HNE modifications. Then, the primary antibody was stripped off and anti-NRF2 antibody was used to detect NRF2 protein as a loading control. [file 2045-3701-2-40-S1.pdf]
